# Genome-Wide Association Study and Selective Sweep Analysis Reveal the Genetic Architecture of Body Weights in a Chicken F_2_ Resource Population

**DOI:** 10.3389/fvets.2022.875454

**Published:** 2022-07-26

**Authors:** Shouzhi Wang, Yuxiang Wang, Yudong Li, Fan Xiao, Huaishun Guo, Haihe Gao, Ning Wang, Hui Zhang, Hui Li

**Affiliations:** ^1^Key Laboratory of Chicken Genetics and Breeding, Ministry of Agriculture and Rural Affairs, Harbin, China; ^2^Key Laboratory of Animal Genetics, Breeding and Reproduction, Education Department of Heilongjiang Province, Harbin, China; ^3^College of Animal Science and Technology, Northeast Agricultural University, Harbin, China; ^4^Fujian Sunnzer Biotechnology Development Co., Ltd., Fujian, China

**Keywords:** broiler, growth trait, body weight, GWAS, selective sweep

## Abstract

Rapid growth is one of the most important economic traits in broiler breeding programs. Identifying markers and genes for growth traits may not only benefit marker-assisted selection (MAS)/genomic selection (GS) but also provide important information for understanding the genetic architecture of growth traits in broilers. In the present study, an F_2_ resource population derived from a cross between the broiler and Baier yellow chicken (a Chinese local breed) was used and body weights from 1 to 12 weeks of age [body weight (BW) 1–BW12)] were measured. A total of 519 F_2_ birds were genome re-sequenced, and a combination of genome-wide association study (GWAS) and selective sweep analysis was carried out to characterize the genetic architecture affecting chicken body weight comprehensively. As a result, 1,539 SNPs with significant effects on body weights at different weeks of age were identified using a genome-wide efficient mixed-model association (GEMMA) package. These SNPs were distributed on chromosomes 1 and 4. Besides, windows under selection identified for BW1–BW12 varied from 1,581 to 2,265. A total of 42 genes were also identified with significant effects on BW1–BW12 based on both GWAS and selective sweep analysis. Among these genes, diacylglycerol kinase eta (*DGKH*), deleted in lymphocytic leukemia (*DLEU7*), forkhead box O17 (*FOXO1*), karyopherin subunit alpha 3 (*KPNA3*), calcium binding protein 39 like (*CAB39L*), potassium voltage-gated channel interacting protein 4 (*KCNIP4*), and slit guidance ligand 2 (*SLIT2*) were considered as important genes for broiler growth based on their basic functions. The results of this study may supply important information for understanding the genetic architecture of growth traits in broilers.

## Introduction

Chicken is one of the most economically important food production animals supplying meat and eggs to human beings. After intensive selection for the past 60 years, substantial advances have been made in improving body weight (BW) in modern commercial meat-type broilers (1). The selection for rapid growth will continue to be one of the most important economic traits in broiler breeding programs. Identifying genetic markers, especially single nucleotide polymorphisms (SNPs), and causal genes affecting BW can provide vital information for marker-assisted selection (MAS) and genomic selection (GS). Additionally, chicken is also considered an essential model for animal genomic studies (2). Therefore, the identification of genomic regions and potential candidate markers/genes can not only help understand the molecular mechanisms involved in the regulation of performance traits in the chicken, but also provide important information for the study in other species.

To date, 4,776 quantitative trait loci (QTLs) for chicken growth traits, including average daily gain and BW at different days of age, are hosted in the Chicken QTL database (release 45) (3). However, many of these QTLs, especially QTLs detected in previous studies, are coarsely mapped, which means that the confidence intervals of these QTLs are large and contain too many genes. The F_2_ design population is beneficial to QTL mapping of traits due to creation of larger genetic variation and trait segregation through the DNA recombination (4). In our previous study, several QTLs for growth and carcass traits were identified using microsatellite markers in the F_2_ resource population from a broiler × a Chinese local breed cross and these QTLs spanned large regions of the genome (5–9).

In the past decade, a genome-wide association study (GWAS) has been used to identify loci significantly associated with traits of interest of domestic animals using high-density SNP panels and genome resequencing technique. In chicken, a host of markers or genes important for growth, meat quality, fertility and so on, were identified in different populations using GWASs (10–16). A GWAS together with a selection signature analysis is widely used to identify SNPs and candidate genes associated with quantitative traits at the genome wide level.

In the present study, birds from an F_2_ resource population that was constructed by crossing broiler cocks derived from Arbor Acres with high abdominal fat content and Baier yellow chicken dams (a Chinese native breed) were genome re-sequenced, and a GWAS together with a selection signature analysis was carried out to comprehensively characterize the genetic architecture affecting BW in chickens. The results of this study can provide important information for understanding the genetic background of growth traits in chickens.

## Materials and Methods

### Animals and Phenotypic Measurements

All animal experiments were conducted according to the guidelines for the Care and Use of Experimental Animals established by the Ministry of Science and Technology of the People's Republic of China (approval number: 2006–398) and approved by the Laboratory Animal Management Committee of Northeast Agricultural University. This study used the F_2_ chicken resource population, which was described previously by Liu et al. (5). To establish this population, we crossed broiler sires derived from a high abdominal fat line divergently selected for abdominal fat with Baier yellow dams (a Chinese native breed). The F_1_ birds were intercrossed to produce an F_2_ population. All F_2_ birds had free access to feed and water. Commercial corn- and soybean-based diets that met all (17) requirements were provided in the study. From hatch to 3 weeks of age, the birds received a starter feed (3,000 kcal of ME/kg and 210 g/kg of CP) and from 3 to 12 weeks of age, the birds were fed a grower diet (3,100 kcal of ME/kg and 190 g/kg of CP) (18). The body weights (BWs) of a total of 519 F_2_ individuals (male and female birds) were measured at hatch and weekly up to 12 weeks of age. Then, quality control of BW was performed. Normality test was conducted to check the distribution of BW at every week using the Shapiro-Wilk test with JMP statistical software version 11.0 (SAS Institute Inc., Cary, NC, USA). If the traits were skewed from the normal test, outlier values were stepwise removed until the traits follow or roughly follow normal distribution. Then, phenotypic data was used for descriptive statistical analysis and GWAS.

### DNA Library Preparation and Sequencing

Total genomic DNA was extracted from the chicken using the reagent test kit. For each bird, a single individual was used for genome sequencing on the Illumina HiSeq PE150 platform with an average depth of 3 ×. Library construction and sample indexing were done as described.

### Population SNP Detection

Paired-end reads were mapped to the GCF_000002315.6_GRCg6a reference genome with Burrows-Wheeler Aligner (Version: 0.7.8) (19). The command line was “BWA mem -t 4 -k 32 –M.” After sorting, the “rmdup” command was used to remove potential PCR duplicates: only the pair with the highest mapping quality was retained, while multiple read pairs had identical external coordinates. After alignment, we performed SNP calling on a population scale with the package SAMtools (20). We then calculated genotype likelihoods from reads for each individual at each genomic location, and the allele frequencies in the sample were determined with a Bayesian approach. The “mpileup” command was used to identify SNPs with the parameters as “-q 1 -C 50 -S -D -m 2 -F 0.002 –u.” Then, to exclude SNP calling errors caused by incorrect mapping, only high-quality SNPs [coverage depth ≥2, root mean square (RMS) mapping quality ≥20, minor allele frequency (MAF) ≥0.05, and miss ≤0.3] were kept for subsequent analysis. After filtering from 15,868,916 raw SNPs, 10,889,955 SNPs remained. The missing genotypes of F_2_ individuals were imputed using 26 sequencing F_0_ individuals with 10-fold. Imputation was performed using BEAGLE 4.0 with default parameter settings (21). The genotypes for each individual were assumed to be unphased, and no relationships between individuals were used. Then, further quality control was conducted (filtered by MAF ≥0.05, missing rate ≤ 0.1, depth ≥2, and LD <0.6). Imputation accuracy (r) was calculated per SNP using the correlation between the observed and imputed genotypes. A total of 7,895,409 SNPs were left after the imputed 10,889,955 SNPs were filtered for the 519 individuals.

### Functional Annotation of Genetic Variants

SNP annotation was performed according to the GCF_000002315.6_GRCg6a reference genome using the package ANNOVAR (Version: 2013-05-20) (22). Based on the genome annotation, SNPs were classified into exonic regions (overlapping with a coding exon), intronic regions (overlapping with an intron), splicing sites (within 2 bp of a splicing junction), upstream and downstream regions (within a 1 kb region upstream or downstream from the transcription start site), and intergenic regions. SNPs in coding exons were further grouped into synonymous SNPs (did not cause amino acid changes) or non-synonymous SNPs (caused amino acid changes). Candidate genes were screened in the 40 kb region upstream and downstream of each top SNP. Besides, mutations causing stop gain and stop loss were also classified into this group. Only the high-quality SNPs were annotated.

### SNP-GWAS

In our association panel containing 519 samples, a total of 7,895,409 SNPs (left and filtered by MAF ≥0.05, missing rate ≤0.1, depth ≥2, and LD <0.6) were used in our GWAS for BW at 1–12 weeks of age (BW1–BW12). Association analysis was conducted using the genome-wide efficient mixed-model association (GEMMA) software package (23). For the mixed linear model analysis, we used the equation:


$$\begin{array}{l} y=S\beta +X\alpha +K\mu +e \end{array}$$

where **y** represents the phenotype; **S** is the incidence matrix of fixed effects and **β** is the vector of corresponding coefficients including the intercept; sex was included as a fixed effect to build up the **S** matrix. **X** represents the vector of SNP genotype and **α** is the corresponding effect of the marker; **K** is incidence matrix for **μ**, **μ** is the vector of random additive genetic effects following the multinormal distribution N (0, $\text{G}{\text{σ}}_{\text{μ}}^{2}$), in which **G** is the genomic relationship matrix based on identity by state (IBS) [Genomic kinship *f*_*ij*_ between individual i and j based on IBS is calculated using the following formula: $f_{ij=}\frac{1}{n}\sum_{k}(g_{ik}-p_{k})(g_{jk}-p_{k})/p_{k}(1-p_{k})$. Where *g*_*ik*_(*g*_*jk*_) is the genotype of the i-th(j-th) bird at the k-th SNP. The frequency *p*_*k*_ is for the major allele and n is the number of SNPs (24)], and ${\text{σ}}_{\text{μ}}^{2}$ is the polygenetic additive variance. **e** represents random residual with a distribution of N (0, $\text{I}{\text{σ}}_{e}^{2}$). We performed principal component analysis (PCA) and tested the significance of top 10 PCAs using the EIGENSTRAT software, and the results showed there are no significant PCs in this population, indicating that there is no striking stratification. Therefore, PCs were not eventually included in the mixed model. The significant level was set as 0.05/N (*P*-value = 6.33 × 10^−9^) to control the genome-wide type 1 error rate and N is the number of informative SNPs.

### Genome-Wide Selective Sweep Analysis

The BWs at each week in 519 birds were ranked. Selection signature analysis of BW was conducted between the two groups (15 birds per group) divided based on the highest and lowest BWs. Using VCFtools (25), we calculated the genome-wide distribution of fixation index (*F*_*ST*_) values and θπ ratios for the defined group pairs (high– and low– BW groups, 40-kb windows sliding in 10-kb steps) to characterize genome-wide selective sweeps related to the selection for growth rate. The θπ ratios were log2-transformed. Subsequently, the empirical percentiles of *F*_*ST*_ and log2 (θπ ratio) in each window were estimated and ranked. The windows with the top 5% *F*_*ST*_ and log2 (θπ ratio) values simultaneously were considered as candidate outliers under strong selective sweeps. All outlier windows were assigned to corresponding SNPs and genes. In other ways, the analysis of the allele frequency differences between the two groups was realized using VCFtools.

## Results

### Descriptive Statistics

The number of animals, means, and standard errors of BW1–BW12 are given in Table 1. Standard deviation (SD) and coefficient of variation (CV) of BWs vary from 10.09 to 392.62 grams and from 13.40 to 18.66%, respectively, indicating that there is large variability in BWs.

**Table 1:** Number of animals (N), mean (M), standard deviation (SD), minimum (MIN), maximum (MAX), and coefficient of variation (CV) of body weight at 1–12 weeks of age (BW1-BW12, in grams) of F_2_ chickens.

**Trait**	**N**	**M**	**SD**	**MIN**	**MAX**	**CV (%)**
BW1	492	75.30	10.09	50.5	102.4	13.40
BW2	490	169.12	22.17	116.1	235.9	13.11
BW3	499	308.70	43.66	170.0	425.0	14.14
BW4	499	479.87	70.48	250.0	660.0	14.69
BW5	489	643.71	94.06	365.0	885.0	14.61
BW6	499	844.44	128.03	480.0	1,160.0	15.16
BW7	504	1,091.51	172.27	660.0	1,535.0	15.78
BW8	500	1,290.74	213.07	790.0	1,845.0	16.51
BW9	493	1,542.36	264.30	945.0	2,185.0	17.14
BW10	504	1,730.23	301.01	1045.0	2,550.0	17.40
BW11	515	1,927.90	350.55	1170.0	2,880.0	18.18
BW12	519	2,104.00	392.62	1240.0	3,185.0	18.66

### GWAS for BW at Different Weeks of Age

**Figure 1:**
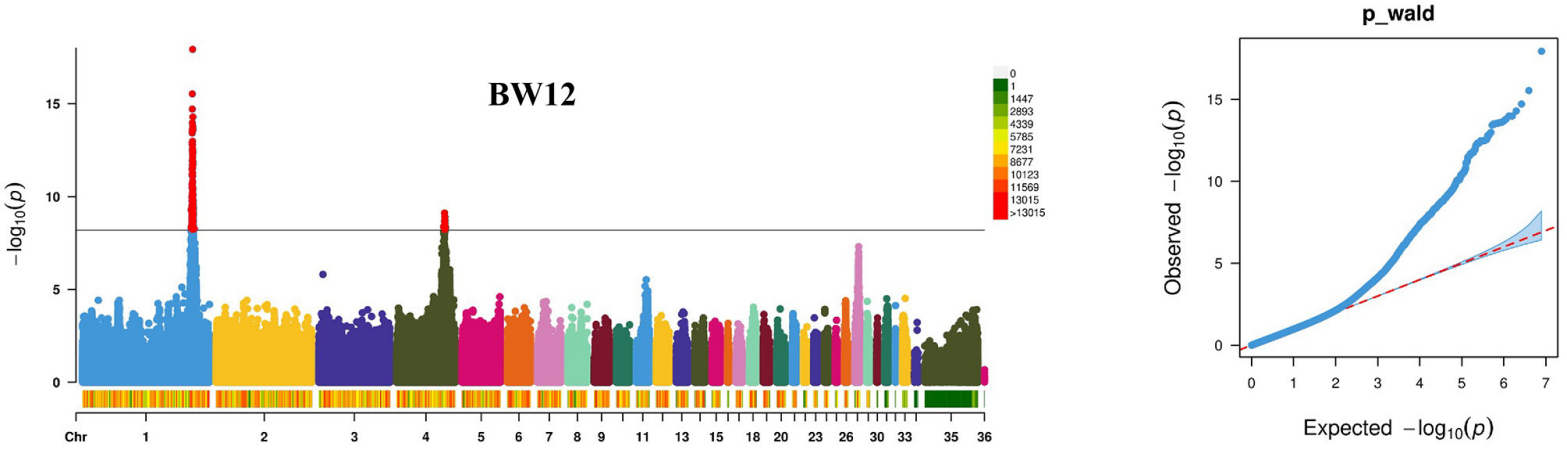
Results of genome-wide association studies for body weight at 12 weeks of age (BW12) using the GEMMA package. The results are presented as the Manhattan plot in the left panel and the Q–Q plot in the right panel. The solid line indicates the threshold to control the genome-wide type I error of 5% (*P* < 6.33 × 10^−9^). The Q-Q plot was used to estimate the difference between observed and expected chi-square statistic values of quantitative traits, indicating that the potential candidate loci related to the traits were not caused by population stratification and the statistical model was reasonable.

The GWAS for BW1–BW12 was carried out using the mixed-model statistical software package GEMMA (23) in 519 individuals of the F_2_ resource population (Figure 1; Supplementary Figure 1). A total of 1,539 SNPs with significant effects (*P* < 6.33 × 10^−9^) on BW1–BW12 were detected (Table 2; Supplementary Table 1). These SNPs with significant effects on BW1–BW12 were distributed on chromosomes 1 and 4. Two lead SNPs responsible for BW12 were detected at the 171,411,019bp (rs316877904) on chromosome 1 (*P* = 8.49e-19), and at the 74,526,009bp (rs13774694) on chromosome 4 (*P* = 3.54e-10), respectively. Linkage disequilibrium (LD) analyses showed that these two lead SNPs were in high LD with some near SNPs (Supplementary Figure 2). Additionally, an interesting phenomenon is that the number of significant SNPs and genes within the region identified on chromosome 1 is consecutively increasing accompanied with weeks of age (from 5 to 12 weeks of age). The genes in 40-kb regions of the SNPs with significant effects on BW1–BW12 were extracted, and 265 genes were detected (Table 2).

**Table 2:** Number of SNPs with significant effects on body weight at different weeks of age and number of selected windows by selective sweep analysis.

**Traits**	**Number of significant SNPs**	**Number of genes detected by GWAS**	**Number of selected windows**	**Number of genes detected by selective sweep analysis**
BW1	0	0	1,581	507
BW2	0	0	1,924	468
BW3	0	0	1,837	499
BW4	0	0	1,842	479
BW5	12	9	1,867	502
BW6	20	15	1,713	429
BW7	79	23	1,973	459
BW8	292	49	1,794	498
BW9	122	30	1,945	523
BW10	203	34	2,020	501
BW11	384	50	2,137	476
BW12	427	55	2,265	538

### Selective Sweep Detected by F_ST_ Combined With Pi Methods

**Figure 2:**
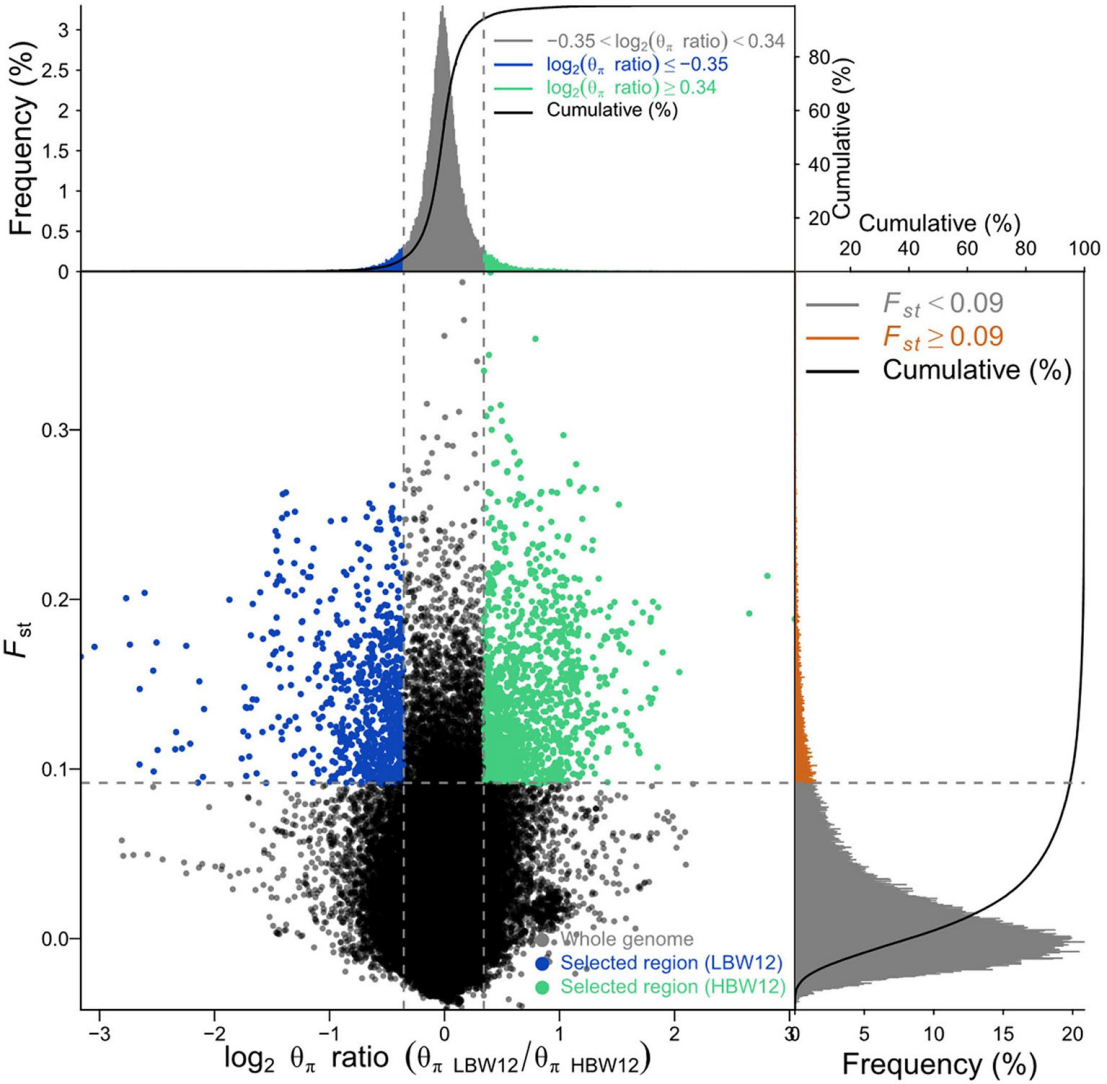
Selective sweep analysis for body weight at 12 weeks of age (BW12) detected by *F*_ST_ and Pi methods. Blue and green colors indicate windows with the top 5% *F*_ST_ and log2 (θπ ratio) values simultaneously, which were considered as the selective sweeps.

We ranked 519 birds according to BW values at every week. The two groups (15 birds per group) were divided in the light of the highest and lowest BWs (Supplementary Table 2). The genome-wide selection signatures were detected using both fixation index (*F*_*ST*_) values and θπ ratios. Windows with the top 5% *F*_*ST*_ and outliers of log2 (θπ ratio) values simultaneously were considered as essential regions under strong selective sweeps. A total of 1,581–2,265 windows under selection were identified for BW1–BW12 (Table 2; Figure 2, Supplementary Figure 3). After deleting the overlaps, 8,554 selected windows were left (Supplementary Table 3). These selection signatures were distributed on nearly all chromosomes with several peaks on chromosomes 1, 4, 5, and Z. All windows under selective sweeps were assigned to corresponding SNPs and genes, and about 429–538 annotated genes were identified. After deleting the overlaps, 2,812 genes were left.

### Candidate Genes for BW at Different Weeks of Age

After compared the GWAS and selection signature gene lists, we found 42 genes overlap (Table 3). Some genes, including diacylglycerol kinase eta (*DGKH*), deleted in lymphocytic leukemia (*DLEU7*), forkhead box O17 (*FOXO1*), karyopherin subunit alpha 3 (*KPNA3*), calcium binding protein 39 like (*CAB39L*), potassium voltage-gated channel interacting protein 4 (*KCNIP4*), and slit guidance ligand 2 (*SLIT2*), were reportedly thought to be necessary for chicken growth based on their basic function study.

**Table 3:** Overlap genes detected by GWAS and selective sweep analysis.

**No**	**Gene**	**Description**	**Location**
1	***DGKH***	diacylglycerol kinase eta	chr1:167536310-167702128
2	*LOC112531567*		chr1:170650308-170655717
3	*LOC112531569*		chr1:171083270-171100945
4	***DLEU7***	deleted in lymphocytic leukemia, 7	chr1:171143861-171152064
5	*SERPINE3*	serpin family E member 3	chr1:171398844-171416703
6	*WDFY2*	WD repeat and FYVE domain containing 2	chr1:171476437-171542083
7	***FOXO1***	forkhead box O1	chr1:171900263-171963540
8	*GTF2F2*	general transcription factor IIF subunit 2	chr1:168988717-169082382
9	*GPALPP1*	GPALPP motifs containing 1	chr1:168950859-168965646
10	*FNDC3A*	fibronectin type III domain containing 3A	chr1:170318524-170431952
11	***KPNA3***	karyopherin subunit alpha 3	chr1:170597160-170650244
12	*FAM124A*	family with sequence similarity 124 member A	chr1:171336721-171377902
13	*SETDB2*	SET domain bifurcated 2	chr1:170526801-170564892
14	*LOC112531568*		chr1:171008615-171039942
15	*MRPS31*	mitochondrial ribosomal protein S31	chr1:171849779-171873661
16	*LHFP*	lipoma HMGIC fusion partner-like 1	chr1:172287762-172427229
17	*NHLRC3*	NHL repeat containing 3	chr1:172554893-172565969
18	*LOC100859822*		chr1:169095074-169099300
19	*SLC25A30*	solute carrier family 25 member 30	chr1:169101373-169110759
20	*COG3*	component of oligomeric golgi complex 3	chr1:169110893-169143086
21	*CDADC1*	cytidine and dCMP deaminase domain containing 1	chr1:170447991-170463866
22	***CAB39L***	calcium binding protein 39 like	chr1:170465092-170526727
23	*ARL11*	ADP ribosylation factor like GTPase 11	chr1:170586604-170597668
24	*LOC107051704*		chr1:170658231-170671446
25	*RNASEH2B*	ribonuclease H2 subunit B	chr1:171220939-171282281
26	*TPTE2*	transmembrane phosphatase with tensin homology	chr1:171808785-171827969
27	*LOC101750153*		chr1:172061063-172091470
28	*COG6*	component of oligomeric golgi complex 6	chr1:172218064-172269404
29	*LOC107052027*		chr1:172532497-172555759
30	*PROSER1*	proline and serine rich 1	chr1:172566162-172586926
31	*LOC107052035*		chr1: 172584113-172597742
32	*C4A*	complement C4A (Rodgers blood group)	chr1:172599919-172652724
33	*FREM2*	FRAS1 related extracellular matrix protein 2	chr1:172657847-172776158
34	*LOC112532426*		chr1:74546421-74555772
35	*RUBCNL*	rubicon like autophagy enhance	chr1:169433550-169453972
36	*LRCH1*	leucine rich repeats and calponin homology domain containing 1	chr1:169520469-169644616
37	*DCLK1*	doublecortin like kinase 1	chr1:174079577-174317535
38	*SMIM20*	small integral membrane protein 20	chr4:73372750-73376776
39	*SLC34A2*	solute carrier family 34 member 2	chr4:73421043-73440963
40	*ANAPC4*	anaphase promoting complex subunit 4	chr4:73476279-73507544
41	***SLIT2***	slit guidance ligand 2	chr4:74981753-75225786
42	***KCNIP4***	potassium voltage-gated channel interacting protein 4	chr4:74568444-74948433

## Discussion

Deciphering of the genetic underpinning of chicken growth traits is conducive to further genetic improvement in breeding program. Owing to the genetic hitch-hiking effect and relatively large QTL confidence intervals of F_2_ population (26), we leveraged an integrative strategy that couples GWAS with selection signatures analysis to dissect the genetic determinants of chicken growth traits at the genome-wide level.

A GWAS has been widely used to identify SNPs and candidate genes associated with important quantitative traits at the genome-wide level. Some important regions associated with production, reproduction, and disease resistance traits in chickens have been identified using GWASs (11, 27–32). Growth trait, especially body weight, is one of the most important economic traits in the poultry industry. Therefore, in this study, we carried out a GWAS for BW from 1 to 12 weeks of age using an F_2_ resource population established by crossing broiler sires with Baier yellow dams. A total of 1,539 SNPs with significant effects on BW1-BW12 were distributed on chromosomes 1 and 4, indicating that BWs are complex traits controlled by multiple genetic determinants. Intriguingly, it could be observed from Supplementary Figure 1 and Table 2 that more significant SNPs were detected over increased ages and BW (from BW5 to BW12). We speculate that there are two possibilities: one is that more genes are likely to be involved in chicken growth and development at the late developmental stages, another is that the involved genes probably play growingly important roles over increased ages. Additionally, according to the numbers of significant SNPs in the region identified on the chromosome 1 for BW5–BW12 (12, 20, 79, 292, 122, 202, 374 and 414, respectively), we found that the top 10 significant SNPs did not change over ages. For instance, 3 SNPs (Chromosome1: 171411019, 171710979, 171925771) are consecutively significant from 5 to 12 weeks; 2 SNPs (Chromosome1: 170926098, 170930631) from 6 to 12 weeks; 5 SNPs (Chromosome1: 170713988, 170714148, 170715097, 170926202, 170926223) from 7 to 12 weeks. These findings reveal that there are shared genetic determinants for BW5–BW12, and these SNP are pleiotropic variants.

To date, an array of QTLs for chicken BW have been identified, and some of them were consistent with the results of the present study. Xu et al. (27) reported that chromosomes 1 and 4 were the two critical chromosomes influencing growth traits, particularly BW in chickens. In the present study, chromosomes 1 and 4 were detected by both GWAS and selective sweep analysis; they were found to harbor important genes for chicken BW. Podisi et al. (33) also reported two significant QTLs for BW at 12 weeks of age on chromosome 1 in broiler cross-bred female chickens. Mebratie et al. (34) carried out a GWAS for BW and found that SNPs with significant effects on BW were located on chicken chromosomes 1, 6, 8, 12, 14, 23, and (35) also identified some SNPs with significant effects on chicken BW located on chromosomes 1, 2, 3, 4, 5, 6, 7, 8, 10, 14, and 21. Chromosome 1 was also identified as harboring important genes for chicken BW in our previous report using the same population by the marker-QTL linkage analysis (6, 8).

As an importantly economic trait, body weight in chickens has undergone long-term artificial selection in the past decades, which is expected to left selective signatures on chicken genomics. The detection of selection signatures can expedite the identification of genes responsible for important economic traits and better understanding the biological mechanisms affected by strong ongoing natural or artificial selection in livestock populations (36, 37). Accordingly, in this study selective signature analysis was utilized to confirm overlapping genomic regions detected by GWAS to screen important candidate genes for chicken BW.

Forty-two annotated genes of chicken were identified by both GWAS and selective sweep analysis. The basic functions of these 42 genes were extracted from the previous reports. Some genes, including *DGKH, DLEU7, FOXO1, KPNA3, CAB39L, KCNIP4, SLIT2*, which were found to be associated with growth traits in farm animals, were considered as important candidate genes for growth traits in broilers. *DGKH* was identified as a candidate gene affecting divergent growth in cattle (38), and this gene could regulate the growth of cattle by regulating the secretion of growth-related hormones (39). *KPNA3* was found to be associated with chicken growth traits in a previous GWAS (10, 40). Zhang et al. (41) identified the *CAB39L* could be a candidate gene for growth and carcass traits by GWAS and pathway enrichment analysis in a Gushi-Anka F_2_ chicken population. The SNPs of *DLEU7* gene were associated with growth traits in Jinghai yellow chickens (40). The replication of *DLEU7* was associated with height in African-derived populations (42). *FOXO1* could influence food intake and then regulate growth (43, 44). *KCNIP4* was identified to be associated with BW of chicken using a GWAS (28). *SLIT2* was also found to be associated with BW at 35 and 41 days of age in chickens (45). Apart from above-mentioned 7 genes reportedly associated with growth traits in farm animals, others 35 genes can be considered as novel candidate genes for chicken growth and development.

Observations at multiple time points for the same individual are called longitudinal traits, which can better describe the growth and production of farm animals than single data records (46). Chicken BWs at different weeks of age are classic longitudinal traits. In the present study GWAS was independently performed for every time point to dissect the genetic basis of BW. A better alternative strategy is to fit the growth curve and then conduct the association analysis using the fitted parameters, which could better mirror growth trajectory and provide novel insight into genetic underpinning of BW in the chicken.

## Conclusions

In summary, in this study, both GWAS and selective sweep analysis were carried out to identify important SNPs and genes for chicken BW. Finally, 42 genes were detected, and some genes, including *DGKH, DLEU7, FOXO1, KPNA3, CAB39L, KCNIP4, SLIT2* were identified as important candidate genes for rapid growth in chickens.

## Data Availability Statement

The datasets presented in this study can be found in online repositories. The names of the repository/repositories and accession number(s) can be found below: [NCBI SRA AND PRJNA861112].

## Ethics Statement

The animal study was reviewed and approved by the Laboratory Animal Management Committee of Northeast Agricultural University. Written informed consent was obtained from the owners for the participation of their animals in this study.

## Author Contributions

SW and YW carried out the experiments, performed the statistical analyses, and prepared the manuscript. YL contributed to the design of the experiments and writing the manuscript. FX, HuG, HaG, and NW contributed to writing the manuscript. HZ and HL conceived and designed the study, participated in data interpretation, and contributed to writing the manuscript. All authors contributed to the article and approved the submitted version.

## Funding

This research was supported by the National Key R&D Program of China (Grant No. 2021YFD1300100), the Joint Guidance Project of Heilongjiang Natural Science Foundation (No. LH2021C036), the National Natural Science Foundation of China (Nos. 31572394 and 31972549), China Agriculture Research System of MOF and MARA (No. CARS-41), and Project of the Ministry of Agriculture and Rural Affairs in China (No. 19190526).

## Conflict of Interest

FX, HsG, and HhG are/were employed by Fujian Sunnzer Biotechnology Development Co., Ltd, China. The remaining authors declare that the research was conducted in the absence of any commercial or financial relationships that could be construed as a potential conflict of interest.

## Publisher's Note

All claims expressed in this article are solely those of the authors and do not necessarily represent those of their affiliated organizations, or those of the publisher, the editors and the reviewers. Any product that may be evaluated in this article, or claim that may be made by its manufacturer, is not guaranteed or endorsed by the publisher.

## References

[1] Hartcher KM Lum HK. Genetic selection of broilers and welfare consequences: a review. Worlds Poult Sci J. (2019) 76:154–67. 10.1080/00439339.2019.168002524397366

[2] Crooijmans RP van Oers PA Strijk JA van der Poel JJ Groenen MA. Preliminary linkage map of the chicken (Gallus domesticus) genome based on microsatellite markers: 77 new markers mapped. Poult Sci. (1996) 75:746–54. 10.3382/ps.0750746 8737840

[3] Hu ZL Park CA Reecy JM. Building a livestock genetic and genomic information knowledgebase through integrative developments of animal QTLdb and CorrDB. Nucleic Acids Res. (2019) 47:D701–10. 10.1093/nar/gky1084 30407520 PMC6323967

[4] Yuan J Sun C Dou T Yi G Qu L Qu L. Identification of promising mutants associated with egg production traits revealed by genome-wide association study. PLoS ONE. (2015) 10:e0140615. 10.1371/journal.pone.0140615 26496084 PMC4619706

[5] Liu X Li H Wang S Hu X Gao Y Wang Q. Mapping quantitative trait loci affecting body weight and abdominal fat weight on chicken chromosome one. Poult Sci. (2007) 86:1084–9. 10.1093/ps/86.6.1084 17495077

[6] Liu X Zhang H Li H Li N Zhang Y Zhang Q. Fine-mapping quantitative trait loci for body weight and abdominal fat traits: effects of marker density and sample size. Poult Sci. (2008) 87:1314–9. 10.3382/ps.2007-00512 18577610

[7] Zhang H Zhang YD Wang SZ Liu XF Zhang Q Tang ZQ. Detection and fine mapping of quantitative trait loci for bone traits on chicken chromosome one. J Anim Breed Genet. (2010) 127:462–8. 10.1111/j.1439-0388.2010.00871.x 21077970

[8] Zhang H Liu SH Zhang Q Zhang YD Wang SZ Wang QG. Fine-mapping of quantitative trait loci for body weight and bone traits and positional cloning of the RB1 gene in chicken. J Anim Breed Genet. (2011) 128:366–75. 10.1111/j.1439-0388.2011.00927.x 21906182

[9] Wang SZ Hu XX Wang ZP Li XC Wang QG Wang YX. Quantitative trait loci associated with body weight and abdominal fat traits on chicken chromosomes 3, 5 and 7. Genet Mol Res. (2012) 11:956–65. 10.4238/2012.April.19.1 22576922

[10] Xie L Luo C Zhang C Zhang R Tang J Nie Q. Genome-wide association study identified a narrow chromosome 1 region associated with chicken growth traits. PLoS ONE. (2012) 7:e30910. 10.1371/journal.pone.0030910 22359555 PMC3281030

[11] Zhang GX Fan QC Wang JY Zhang T Xue Q Shi HQ. Genome-wide association study on reproductive traits in Jinghai yellow chicken. Anim Reprod Sci. (2015) 163:30–4. 10.1016/j.anireprosci.2015.09.011 26498507

[12] Zhang T Fan QC Wang JY Zhang GX Gu YP Tang Y. Genome-wide association study of meat quality traits in chicken. Genet Mol Res. (2015) 14:10452–60. 10.4238/2015.September.8.6 26400276

[13] Psifidi A Banos G Matika O Desta TT Bettridge J Hume DA. Genome-wide association studies of immune, disease and production traits in indigenous chicken ecotypes. Genet Sel Evol. (2016) 48:74. 10.1186/s12711-016-0252-7 27687164 PMC5041578

[14] Raeesi V Ehsani A Torshizi RV Sargolzaei M Masoudi AA Dideban R. Genome-wide association study of cell-mediated immune response in chicken. J Anim Breed Genet. (2017) 134:405–11. 10.1111/jbg.12265 28295717

[15] Moreira G Boschiero C Cesar A Reecy JM Godoy TF Pértille F. Integration of genome wide association studies and whole genome sequencing provides novel insights into fat deposition in chicken. Sci Rep. (2018) 8:16222. 10.1038/s41598-018-34364-0 30385857 PMC6212401

[16] Liu Z Yang N Yan Y Li G Liu A Wu G. Genome-wide association analysis of egg production performance in chickens across the whole laying period. BMC Genet. (2019) 20:67. 10.1186/s12863-019-0771-7 31412760 PMC6693279

[17] National Research Council. Nutrient Requirements of Poultry. Washington, DC: Natl Acad Press (1994).

[18] Wang Q Li H Li N Leng L Wang Y Tang Z. Identification of single nucleotide polymorphism of adipocyte fatty acid-binding protein gene and its association with fatness traits in the chicken. Poult Sci. (2006) 85:429–34. 10.1093/ps/85.3.429 16553271

[19] Li H Durbin R. Fast and accurate short read alignment with Burrows-Wheeler transform. Bioinformatics. (2009) 25:1754–60. 10.1093/bioinformatics/btp324 19451168 PMC2705234

[20] Li H Handsaker B Wysoker A Fennell T Ruan J Homer N. The sequence alignment/map format and SAMtools. Bioinformatics. (2009) 25:2078–9. 10.1093/bioinformatics/btp352 19505943 PMC2723002

[21] Browning BL Browning SR. A unified approach to genotype imputation and haplotype-phase inference for large data sets of trios and unrelated individuals. Am J Hum Genet. (2009) 84:210–23. 10.1016/j.ajhg.2009.01.005 19200528 PMC2668004

[22] Wang K Li M Hakonarson H. ANNOVAR: functional annotation of genetic variants from high-throughput sequencing data. Nucleic Acids Res. (2010) 38:e164. 10.1093/nar/gkq603 20601685 PMC2938201

[23] Zhou X Stephens M. Genome-wide efficient mixed-model analysis for association studies. Nat Genet. (2012) 44:821–4. 10.1038/ng.2310 22706312 PMC3386377

[24] Nagamine Y Pong-Wong R Navarro P Vitart V Hayward C Rudan I. Localising loci underlying complex trait variation using regional genomic relationship mapping. PLoS ONE. (2012) 7:e46501. 10.1371/journal.pone.0046501 23077511 PMC3471913

[25] Danecek P Auton A Abecasis G Albers CA Banks E DePristo MA. 1000 Genomes Project Analysis Group. The variant call format and VCFtools. Bioinformatics. (2011) 27:2156–8. 10.1093/bioinformatics/btr330 21653522 PMC3137218

[26] Johnsson M Rubin CJ Höglund A Sahlqvist AS Jonsson KB Kerje S. The role of pleiotropy and linkage in genes affecting a sexual ornament and bone allocation in the chicken. Mol Ecol. (2014) 23:2275–86. 10.1111/mec.12723 24655072

[27] Xu Z Nie Q Zhang X. Overview of genomic insights into chicken growth traits based on genome-wide association study and microRNA Regulation. Curr Genomics. (2013) 14:137–46. 10.2174/1389202911314020006 24082823 PMC3637678

[28] Jin CF Chen YJ Yang ZQ Shi K Chen CK. A genome-wide association study of growth trait-related single nucleotide polymorphisms in Chinese Yancheng chickens. Genet Mol Res. (2015) 14:15783–92. 10.4238/2015.December.1.30 26634546

[29] Reyer H Hawken R Murani E Ponsuksili S Wimmers K. The genetics of feed conversion efficiency traits in a commercial broiler line. Sci Rep. (2015) 5:16387. 10.1038/srep16387 26552583 PMC4639841

[30] Yuan J Wang K Yi G Ma M Dou T Sun C. Genome-wide association studies for feed intake and efficiency in two laying periods of chickens. Genet Sel Evol. (2015) 47:82. 10.1186/s12711-015-0161-1 26475174 PMC4608132

[31] Wang W Zhang T Wang J Zhang G Wang Y Zhang Y. Genome-wide association study of 8 carcass traits in Jinghai yellow chickens using specific-locus amplified fragment sequencing technology. Poult Sci. (2016) 95:500–6. 10.3382/ps/pev266 26614681 PMC4957485

[32] Fan QC Wu PF Dai GJ Zhang GX Zhang T Xue Q. Identification of 19 loci for reproductive traits in a local Chinese chicken by genome-wide study. Genet Mol Res. (2017) 16:gmr16019431. 10.4238/gmr16019431 28340264

[33] Podisi BK Knott SA Burt DW Hocking PM. Comparative analysis of quantitative trait loci for body weight, growth rate and growth curve parameters from 3 to 72 weeks of age in female chickens of a broiler-layer cross. BMC Genet. (2013) 14:22. 10.1186/1471-2156-14-22 23496818 PMC3606837

[34] Mebratie W Reyer H Wimmers K Bovenhuis H Jensen J. Genome wide association study of body weight and feed efficiency traits in a commercial broiler chicken population, a re-visitation. Sci Rep. (2019) 9:922. 10.1038/s41598-018-37216-z 30696883 PMC6351590

[35] Van Goor A Bolek KJ Ashwell CM Persia ME Rothschild MF Schmidt CJ. Identification of quantitative trait loci for body temperature, body weight, breast yield, and digestibility in an advanced intercross line of chickens under heat stress. Genet Sel Evol. (2015) 47:96. 10.1186/s12711-015-0176-7 26681307 PMC4683778

[36] Li D Huang M Zhuang Z Ding R Gu T Hong L. Genomic analyses revealed the genetic difference and potential selection genes of growth traits in two duroc lines. Front Vet Sci. (2021) 8:725367. 10.3389/fvets.2021.725367 34557543 PMC8453014

[37] Liu D Chen Z Zhao W Guo L Sun H Zhu K. Genome-wide selection signatures detection in Shanghai Holstein cattle population identified genes related to adaption, health and reproduction traits. BMC Genomics. (2021) 22:747. 10.1186/s12864-021-08042-x 34654366 PMC8520274

[38] Widmann P Reverter A Fortes MR Weikard R Suhre K Hammon H. A systems biology approach using metabolomic data reveals genes and pathways interacting to modulate divergent growth in cattle. BMC Genomics. (2013) 14:798. 10.1186/1471-2164-14-798 24246134 PMC3840609

[39] Lu X Arbab A Zhang Z Fan Y Han Z Gao Q. Comparative transcriptomic analysis of the pituitary gland between cattle breeds differing in growth: yunling cattle and leiqiong cattle. Animals. (2020) 10:1271. 10.3390/ani10081271 32722439 PMC7460210

[40] Abdalhag MA Zhang T Fan QC Zhang XQ Zhang GX Wang JY. Single nucleotide polymorphisms associated with growth traits in Jinghai yellow chickens. Genet Mol Res. (2015) 14:16169–77. 10.4238/2015.December.8.6 26662409

[41] Zhang Y Wang Y Li Y Wu J Wang X Bian C. Genome-wide association study reveals the genetic determinism of growth traits in a Gushi-Anka F_2_ chicken population. Heredity. (2021) 126:293–307. 10.1038/s41437-020-00365-x 32989280 PMC8026619

[42] Kang SJ Chiang CW Palmer CD Tayo BO Lettre G Butler JL. Genome-wide association of anthropometric traits in African- and African-derived populations. Hum Mol Genet. (2010) 19:2725–38. 10.1093/hmg/ddq154 20400458 PMC2883343

[43] Zhu C Xu P He Y Yuan Y Wang T Cai X. Heparin increases food intake through AgRP neurons. Cell Rep. (2017) 20:2455–67. 10.1016/j.celrep.2017.08.049 28877477 PMC6310124

[44] Sasaki T Kitamura T. Roles of FoxO1 and Sirt1 in the central regulation of food intake. Endocr J. (2010) 57:939–46. 10.1507/endocrj.k10e-320 21048357

[45] Pértille F Zanella R Felício AM Ledur MC Peixoto JO Coutinho LL. Identification of polymorphisms associated with production traits on chicken (Gallus gallus) chromosome 4. Genet Mol Res. (2015) 14:10717–28. 10.4238/2015.September.9.11 26400301

[46] Duan X An B Du L Chang T Liang M Yang BG. Genome-wide association analysis of growth curve parameters in Chinese simmental beef cattle. Animals. (2021) 11:192. 10.3390/ani11010192 33467455 PMC7830728

