# Supplementary material for: Genome-Wide Association Study and Selective Sweep Analysis Reveal the Genetic Architecture of Body Weights in a Chicken F2 Resource Population
Source: Front Vet Sci. 2022 Jul 26;9:875454. doi: 10.3389/fvets.2022.875454 (PMC9361851; doi:10.3389/fvets.2022.875454)
Supplement: Supplementary file 2 [file Table_2.DOCX]

Table S2 The number of male birds and female birds in every group for body weight at week

| Traits | Group high BW | | Group low BW | |
| --- | --- | --- | --- | --- |
|  | No. male birds | No. female birds | No. male birds | No. female birds |
| BW1 | 9 | 6 | 6 | 9 |
| BW2 | 12 | 3 | 5 | 10 |
| BW3 | 14 | 1 | 1 | 14 |
| BW4 | 15 | 0 | 1 | 14 |
| BW5 | 15 | 0 | 1 | 14 |
| BW6 | 15 | 0 | 2 | 13 |
| BW7 | 15 | 0 | 2 | 13 |
| BW8 | 15 | 0 | 2 | 13 |
| BW9 | 15 | 0 | 1 | 14 |
| BW10 | 15 | 0 | 1 | 14 |
| BW11 | 15 | 0 | 1 | 14 |
| BW12 | 15 | 0 | 1 | 14 |
