# Supplementary figures and images for: Genome-Wide Association Study and Selective Sweep Analysis Reveal the Genetic Architecture of Body Weights in a Chicken F2 Resource Population
Source: Front Vet Sci. 2022 Jul 26;9:875454. doi: 10.3389/fvets.2022.875454 (PMC9361851; doi:10.3389/fvets.2022.875454)

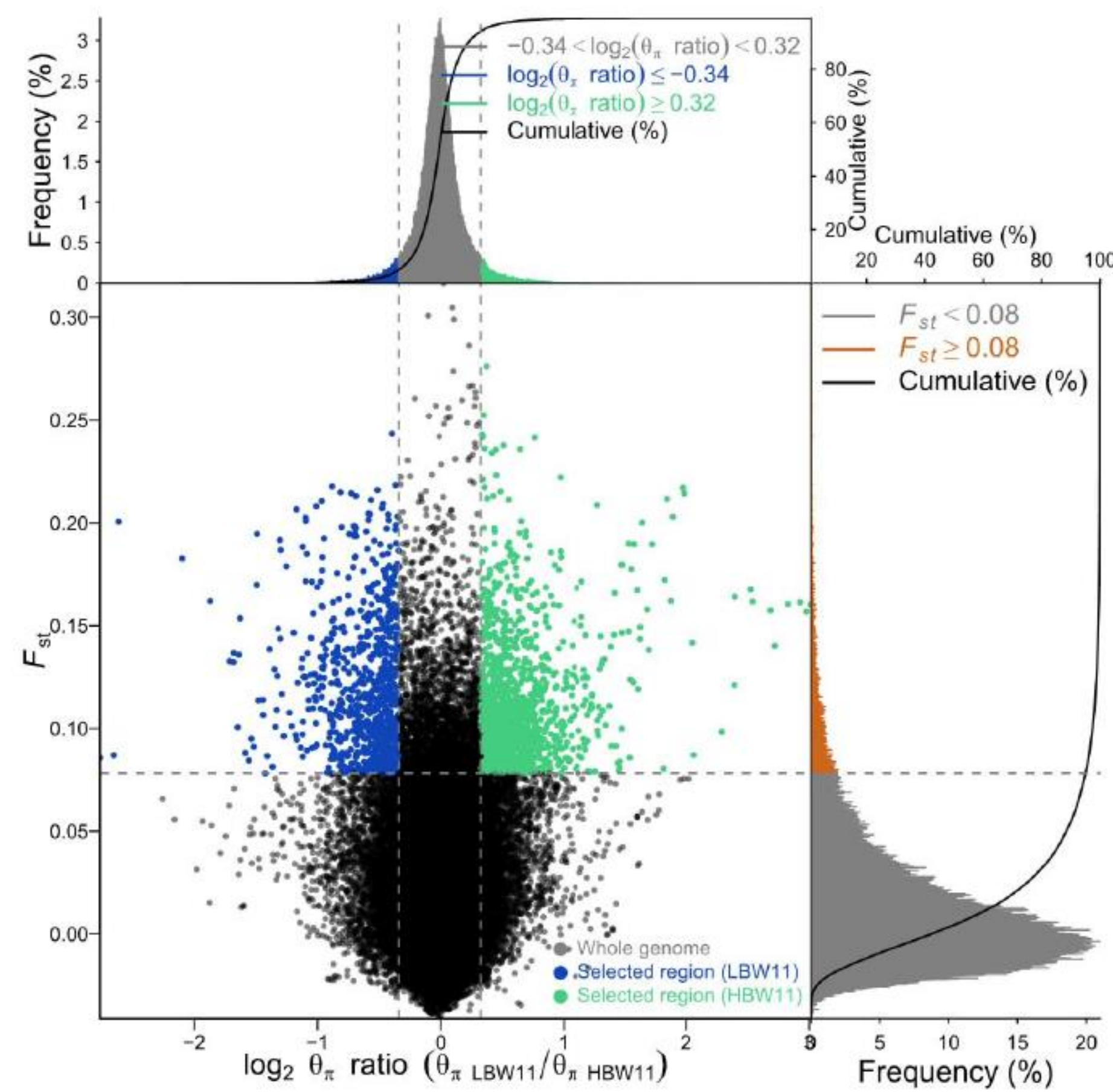

Supplement: Supplementary file 6 [file Data_Sheet_3.PDF]
