# Supplementary material for: Genome-Wide Association Study and Selective Sweep Analysis Reveal the Genetic Architecture of Body Weights in a Chicken F2 Resource Population
Source: Front Vet Sci. 2022 Jul 26;9:875454. doi: 10.3389/fvets.2022.875454 (PMC9361851; doi:10.3389/fvets.2022.875454)
Supplement: Supplementary file 4 [file Data_Sheet_1.PDF]

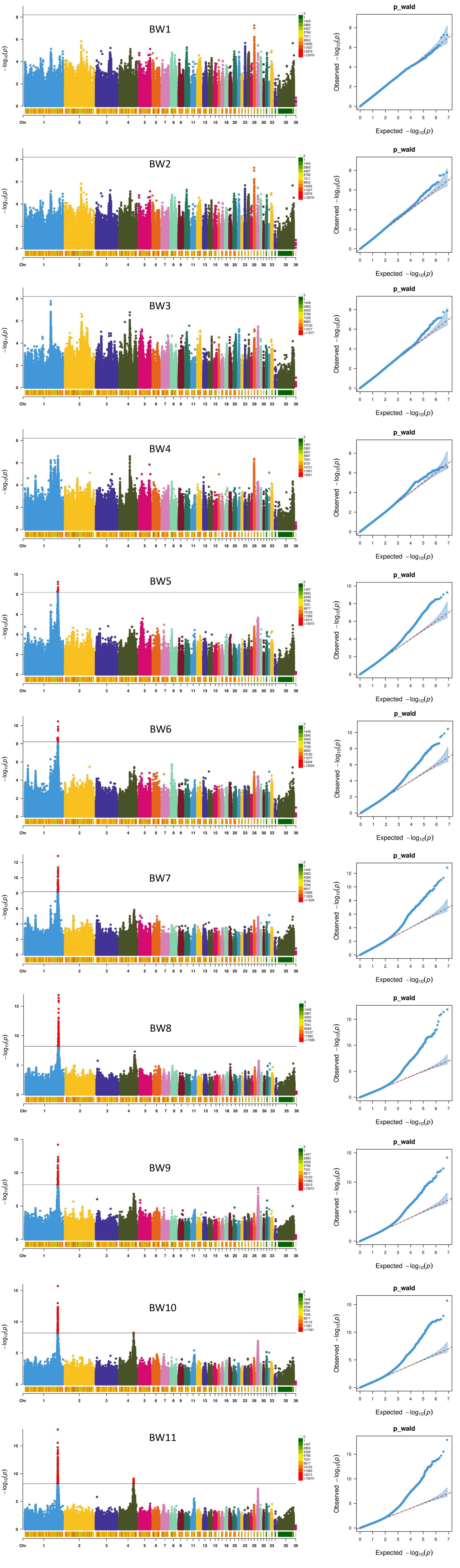
BW1
BW2
BW3
BW4
BW5
BW6
BW7
BW8
BW9
BW10
BW11
$-\log_{10}(p)$
Chr
p_wald
Observed $-\log_{10}(p)$
Expected $-\log_{10}(p)$
