# Supplementary material for: Genome-Wide Association Study and Selective Sweep Analysis Reveal the Genetic Architecture of Body Weights in a Chicken F2 Resource Population
Source: Front Vet Sci. 2022 Jul 26;9:875454. doi: 10.3389/fvets.2022.875454 (PMC9361851; doi:10.3389/fvets.2022.875454)
Supplement: Supplementary file 5 [file Data_Sheet_2.PDF]

BW5

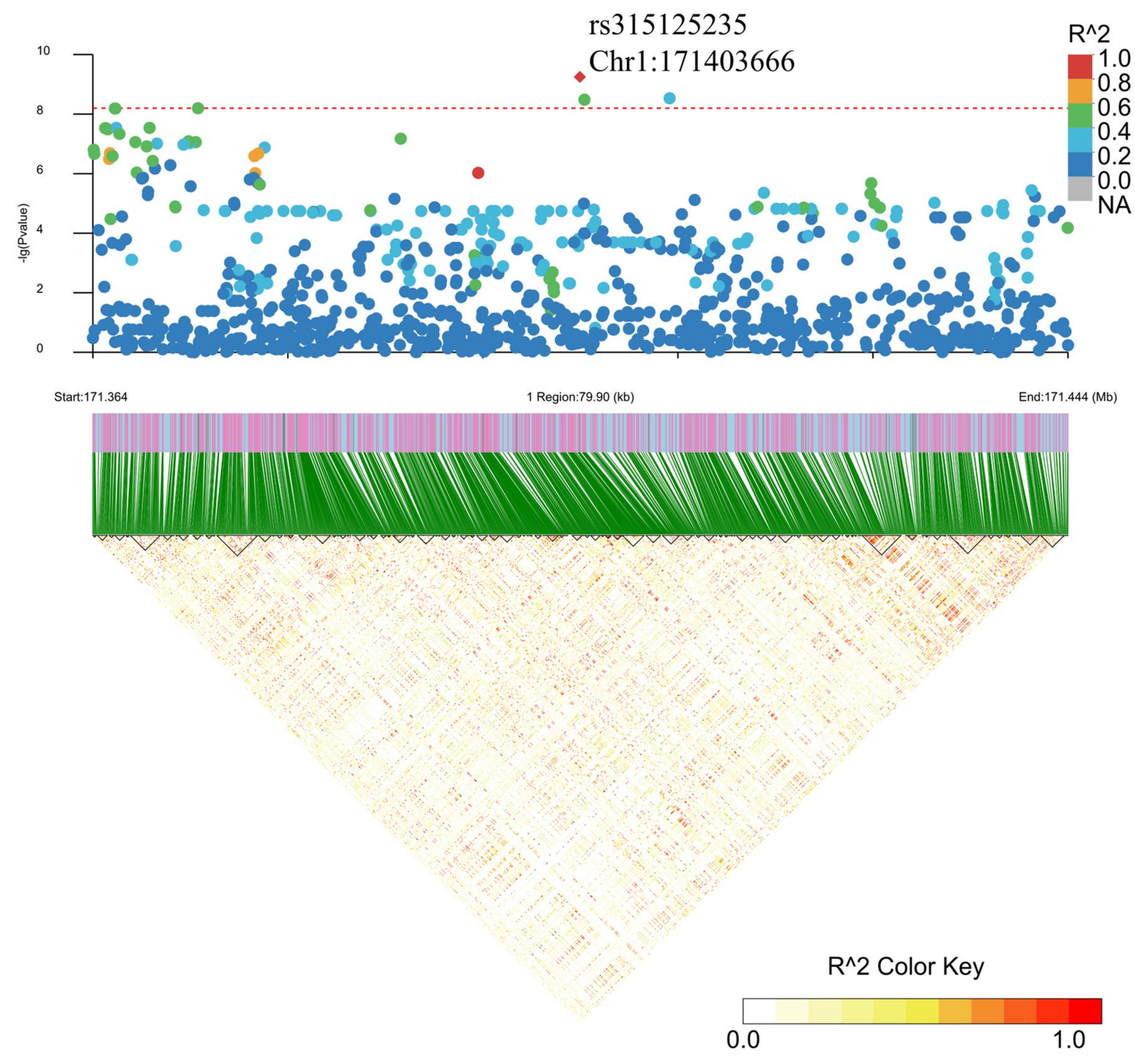

BW6

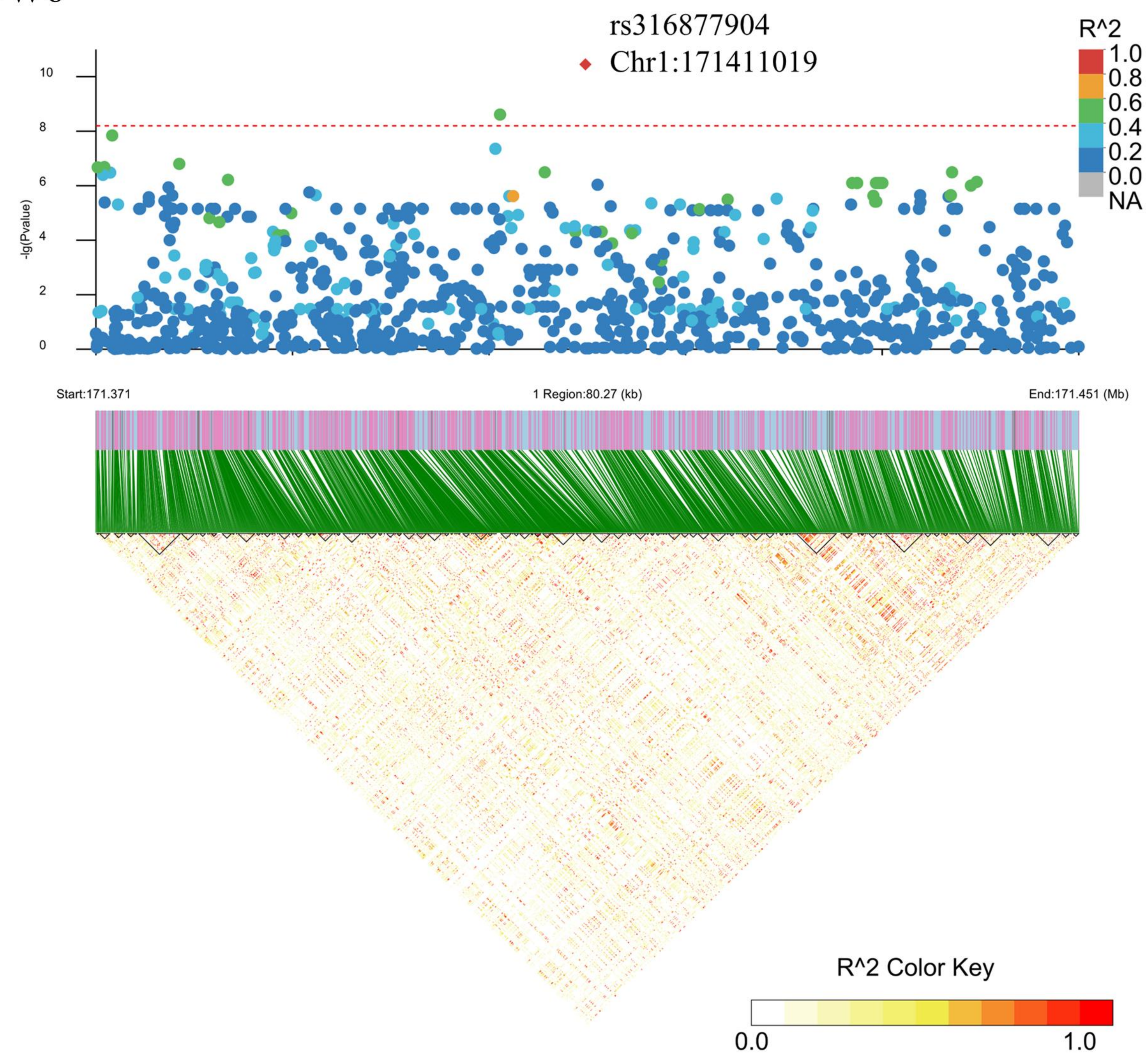

BW7

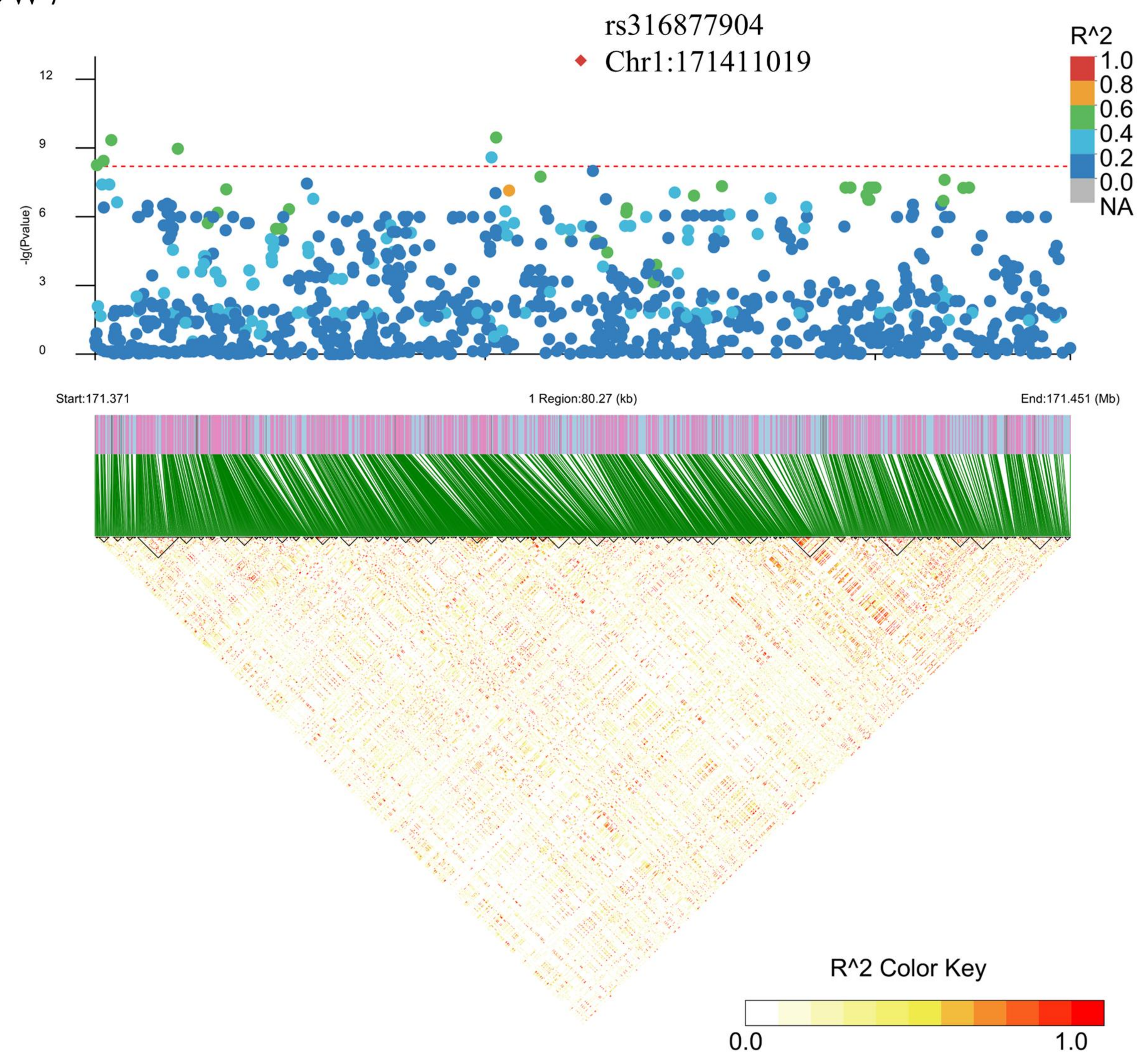

BW8

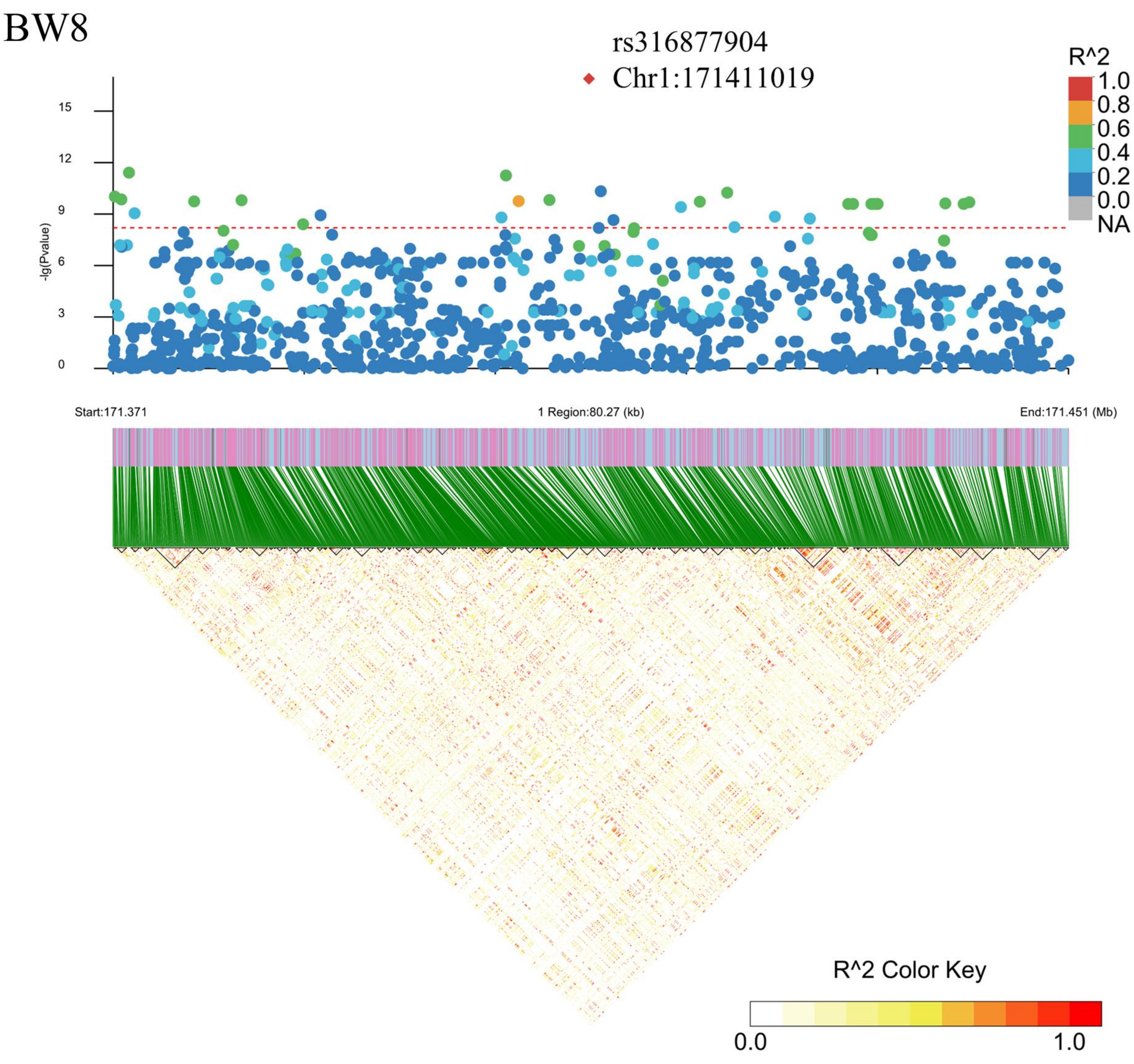

BW9

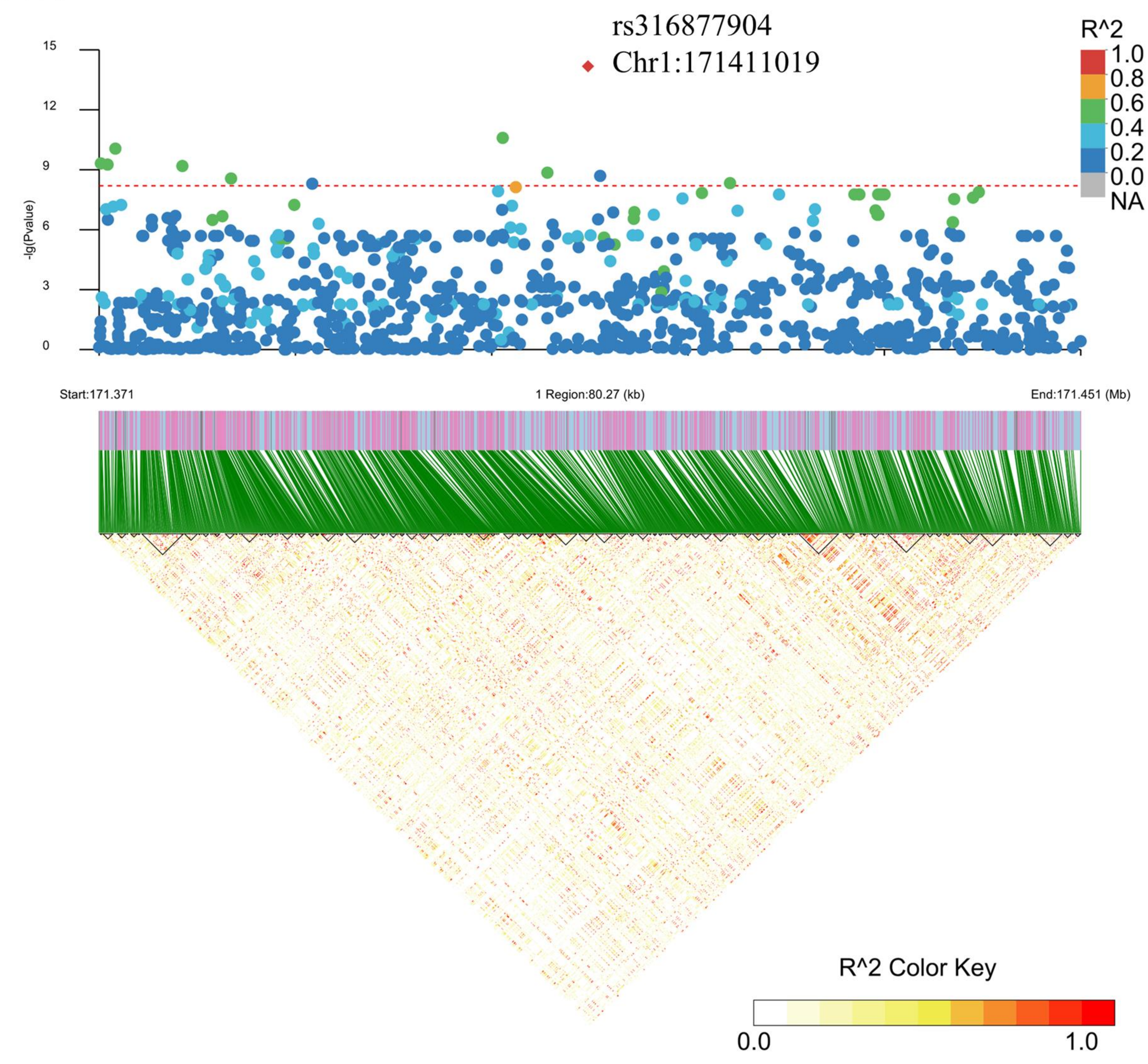

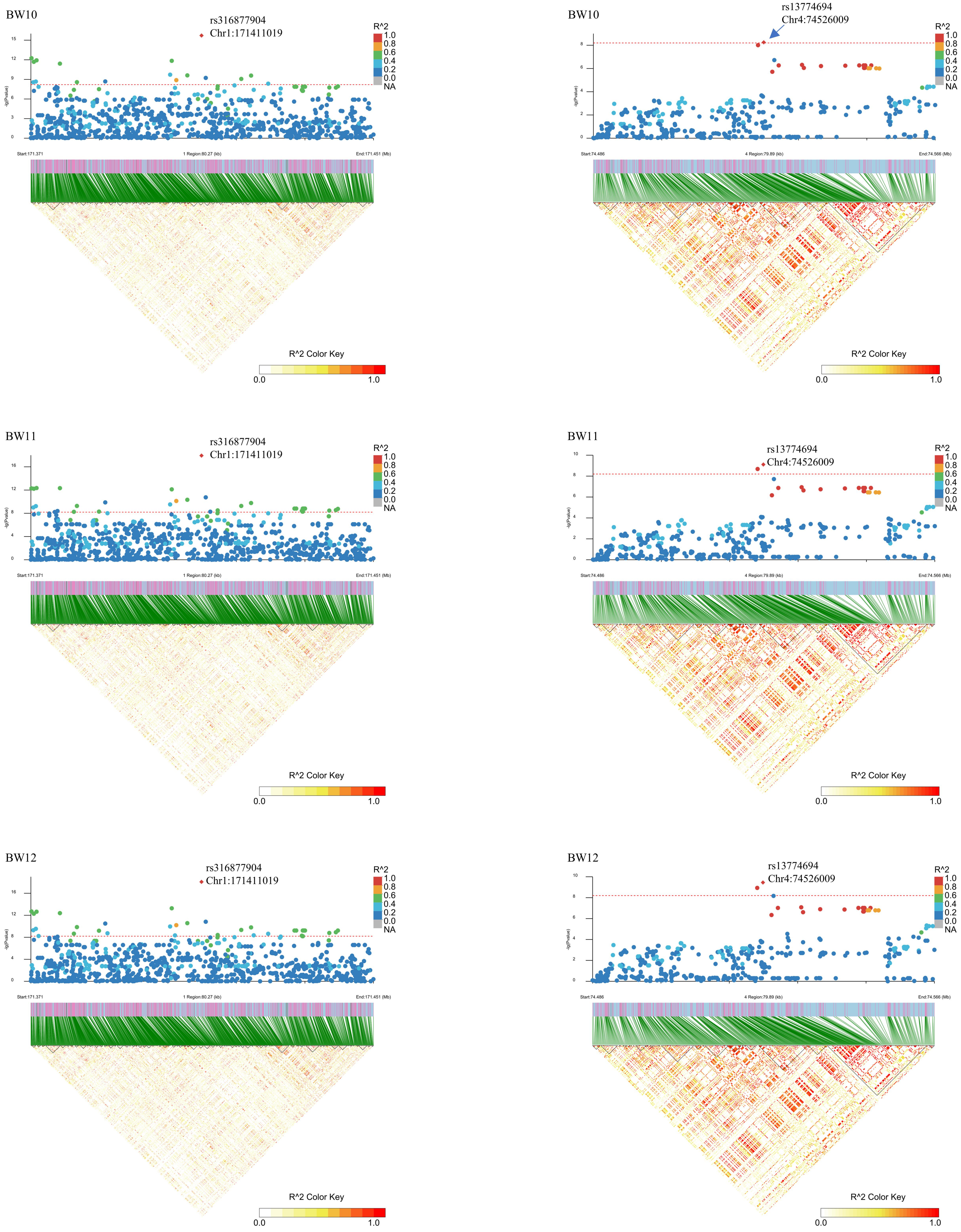

BW10
rs316877904
Chr1:171411019
R^2
1.0
0.8
0.6
0.4
0.2
0.0
NA
-lg(Pvalue)
Start:171.371
1 Region:80.27 (kb)
End:171.451 (Mb)
R^2 Color Key
0.0
1.0
BW10
rs13774694
Chr4:74526009
Start:74.486
4 Region:79.89 (kb)
End:74.566 (Mb)
BW11
rs316877904
Chr1:171411019
BW11
rs13774694
Chr4:74526009
BW12
rs316877904
Chr1:171411019
BW12
rs13774694
Chr4:74526009
